# Apomorphine Reduces A53T α-Synuclein-Induced Microglial Reactivity Through Activation of NRF2 Signalling Pathway

**DOI:** 10.1007/s10571-021-01131-1

**Published:** 2021-08-20

**Authors:** Tony Heurtaux, Melanie Kirchmeyer, Eric Koncina, Paul Felten, Lorraine Richart, Oihane Uriarte Huarte, Herve Schohn, Michel Mittelbronn

**Affiliations:** 1 Faculty of Science, Technology and Medicine, University of Luxembourg, L-4365 Esch-sur-Alzette, Luxembourg; 2 Department of Life Sciences and Medicine (DLSM), University of Luxembourg, 7, Avenue des Hauts Fourneaux, L-4362 Esch-sur-Alzette, Luxembourg; 3 Luxembourg Center of Neuropathology (LCNP), L-3555 Dudelange, Luxembourg; 4 Department of Oncology (DONC), Luxembourg Institute of Health (LIH), L-1526 Strassen, Luxembourg; 5 Luxembourg Centre for Systems Biomedicine (LCSB), University of Luxembourg, L-4362 Esch-sur-Alzette, Luxembourg; 6 CNRS, CRAN, Université de Lorraine, 54000 Nancy, France; 7 National Center of Pathology (NCP), Laboratoire National de Santé (LNS), L-3555 Dudelange, Luxembourg

**Keywords:** Primary microglia, Mutant alpha-synuclein, Inflammation, Apomorphine, Transcription factor recruitment

## Abstract

The chiral molecule, apomorphine, is currently used for the treatment of Parkinson’s disease (PD). As a potent dopamine receptor agonist, this lipophilic compound is especially effective for treating motor fluctuations in advanced PD patients. In addition to its receptor-mediated actions, apomorphine has also antioxidant and free radical scavenger activities. Neuroinflammation, oxidative stress, and microglia reactivity have emerged as central players in PD. Thus, modulating microglia activation in PD may be a valid therapeutic strategy. We previously reported that murine microglia are strongly activated upon exposure to A53T mutant α-synuclein. The present study was designed to investigate whether apomorphine enantiomers could modulate this A53T-induced microglial activation. Taken together, the results provided evidence that apomorphine enantiomers decrease A53T-induced microgliosis, through the activation of the NRF2 signalling pathway, leading to a lower pro-inflammatory state and restoring the phagocytic activity. Suppressing NRF2 recruitment (trigonelline exposure) or silencing specifically *Nfe2l2* gene (siRNA treatment) abolished or strongly decreased the anti-inflammatory activity of apomorphine. In conclusion, apomorphine, which is already used in PD patients to mimic dopamine activity, may also be suitable to decrease α-synuclein-induced microglial reactivity.

## Introduction

Parkinson’s disease (PD) is the second most important neurological disorder, following Alzheimer's disease and related dementias. It has been estimated that 7–10 million people worldwide are affected by this disease. PD is clinically characterized by motor symptoms such as tremor, bradykinesia, muscle stiffness, postural instability, gait difficulties and vocal symptoms, but also by additional non-motor symptoms comprising smell and gastrointestinal dysfunction, depression, anxiety, psychosis and cognitive changes (Kalia and Lang 2015). Histologically, PD is an age-related disorder characterized by a progressive loss of neurons in the pars compacta of the Substantia Nigra (Cuenca et al. 2019). These neurons produce dopamine, a neurotransmitter that helps to regulate movement. Different strategies have been implemented in order to compensate for this lack of dopamine: dopamine replacement therapy (levodopa), stimulation of dopamine receptors (dopamine agonists) and other options using monoamine oxidase-B (MAO-B), DOPA decarboxylase (AADC) or catechol-O-methyltransferase (COMT) inhibitors implicated in dopamine metabolism. The first dopamine agonist, apomorphine, has been discovered by Matthiessen and Wright in the mid-1800s. However, it was only in the mid-1900s that the structural similarity between apomorphine and dopamine has been established and that first trials in PD patients have been reported (Schwab et al. 1951). Due to its low bioavailability, short duration of action and side-effects, apomorphine has been replaced by levodopa (Boyle and Ondo 2015; Jenner and Katzenschlager 2016). However, levodopa monotherapy presented some limitations as advanced stage PD patients suffer from motor and non-motor complications (Rascol et al. 2003; LeWitt and Fahn 2016). In combination with levodopa, subcutaneous infusions of apomorphine partially alleviated these persistent motor complications, but resulted in a pronounced improvement in the time periods when therapy was no longer as effective (Boyle and Ondo 2015; Katzenschlager et al. 2018; Pessoa et al. 2018; Antonini and Jenner 2018).

Based on its physicochemical properties, apomorphine is a chiral molecule existing as two enantiomers: R- and S-apomorphine. The R-isomer of apomorphine is a dopamine D1/D2 receptor agonist, acting both pre- and post-synaptically, whereas the S-isomer is devoid of dopamine receptor agonist activities. Previous studies suggest that apomorphine might be neuroprotective (Grünblatt et al. 2001; Hara et al. 2006; Mead et al. 2013). Both enantiomers also possess antioxidant activity governed by the activation of the Nuclear factor E2-related factor 2/Antioxidant response element (NRF2/ARE) signalling pathway (Hara et al. 2006; Mead et al. 2013). The NRF2 transcription factor is known to regulate a vast array of genes (Papp et al. 2012; Tonelli et al. 2018) and to be linked to beneficial but also deleterious effects in health disorders (Hybertson and Gao 2014; Cuadrado et al. 2018). The regulation of NRF2 activities is the subject of extensive research. Controlling inflammation is one of the crucial roles of NRF2. It has been indeed reported that recruiting the NRF2 transcription factor can decrease neuroinflammation (Innamorato et al. 2008; Kobayashi et al. 2016; Han et al. 2019; Sivandzade et al. 2019a) whereas attenuating it does the opposite and might disturb brain homeostasis (Sandberg et al. 2014; Tarantini et al. 2018; Liu et al. 2020). In the last 5 years, many studies highlighted NRF2 as a potential therapeutic target to treat neurodegenerative diseases (Lastres-Becker et al. 2016; Skibinski et al. 2017; Deshmukh et al. 2017; Dinkova-Kostova et al. 2018; Petrillo et al. 2020; Brandes and Gray 2020). A likely role for neuroinflammation in PD has been reported (Kempuraj et al. 2017; Gelders et al. 2018; Troncoso-Escudero et al. 2018). Clinical and preclinical evidence linking neuroinflammation to PD have been described in postmortem collected brain tissues, blood or cerebrospinal fluid samples as well as in transgenic or neurotoxin-based animal models (McGeer et al. 2001; Perry 2012; Gelders et al. 2018). Elevated levels of pro-inflammatory cytokines/chemokines, reactive oxygen/nitrogen species (ROS/RNS) are found to be increased in these PD-related biological samples.

PD is also characterized by the presence of proteinaceous insoluble inclusions, called Lewy bodies, containing a high concentration of α-synuclein (α-syn) (Spillantini et al. 1997). Its accumulation causes the death of dopaminergic neurons and leads to the loss of the nigrostriatal pathway (Lundblad et al. 2012). While most of PD cases are sporadic, mutations of the gene encoding the α-syn protein (*Snca*), cause inherited forms of PD (Polymeropoulos et al. 1997; Schneider and Alcalay 2017; Lunati et al. 2018). Pathological mutated or aggregated α-synuclein exacerbates the progression of PD through microglial activation (Tang and Le 2016; Zhang et al. 2017; Heneka et al. 2018; Duffy et al. 2018; Grozdanov et al. 2019). In a previous study, we reported that wild-type α-synuclein (WT) and three well-characterized mutant forms (A30P, A53T and E46K) differentially activate primary mouse microglial cultures (Hoenen et al. 2016). Indeed, exposure to A53T was able to increase the pro-inflammatory phenotype in comparison to the other α-synuclein proteins (A53T > A30P > WT > E46K).

Microglia are the tissue-resident macrophages of the brain. This complex population of immune cells is the first line of defence to all types of CNS disorders. Reactive microglia, through various pattern recognition receptors, are known to secrete high levels of pro-inflammatory mediators, growth factors, cell surface molecules as well as reactive species (ROS/RNS). The increase in these neurotoxic molecules promotes neuroinflammation and neurodegeneration (Kempuraj et al. 2016; Chen et al. 2016). To maintain a healthy tissue environment, microglia express a large variety of receptors for cytokines, chemokines, neurohormones, neuromodulators and neurotransmitters (Harry 2013). The presence of dopaminergic receptors on microglia was first established by Färber and collaborators (Färber et al. 2005). There is increasing evidence that chronic stimulation of neurotransmitter receptors may attenuate microglial activation (Färber et al. 2005; Dominguez-Meijide et al. 2017). Therefore, we explored whether apomorphine enantiomers are able to alleviate microglial cells by reducing the pro-inflammatory state induced by an A53T mutant α-synuclein exposure. For the first time, our study shows that in addition to be a dopamine receptor agonist, apomorphine plays a beneficial role by reducing microglial reactivity in a PD-like context through the activation of the NRF2-signalling pathway.

## Materials and Methods

### Ethics Statement

The Animal Experimentation Ethics Committee (AEEC) of the University of Luxembourg and the relevant government agencies approved our animal experiments. Besides, all the procedures were performed following the 2010/63/EU European Union Directive.

### Alpha-Synuclein

Purified recombinant human α-synuclein (A53T mutant protein; AJ Roboscreen GmbH, Germany) was resuspended in sterile H_2_O to reach a final concentration of 100 µM. Endotoxin contamination was assessed with the PYROGENT™ Plus Gel Clot LAL Single Test Vials kit (Lonza, Belgium). Briefly, this test is based on a visual inspection of a gel clot formation at 37 °C due to the presence of endotoxins. A lipopolysaccharide standard curve determined the sensitivity of the LAL kit at 0.01 ng/ml (0.03 U/ml according to the provider’s datasheet). A53T α-synuclein aliquots (100 µM) were stored at − 20 °C until use.

### Primary Microglia Cultures and Treatments

Mixed glial cell cultures were derived from the brains of newborn C57BL/6J mice (Harlan, The Netherlands) (Losciuto et al. 2012). Since the number of extracted microglia cells from one newborn mouse brain only ranged between 0.5 and 0.7 million cells (0.6 million in average), we had to pool microglial cells from multiple newborn mice before starting the respective experiments. For the current study, a total number of 300 million microglial cells was used deriving from an approximated equivalent of 500 mice. Meninges and large blood vessels were removed. Brains were then minced and mechanically dissociated in phosphate-buffered saline (PBS) solution. Afterwards, cells were plated in culture medium composed of Dulbecco’s Modified Eagle Medium (DMEM), 10% (v/v) fetal bovine serum (FBS), 100 U/ml penicillin and 100 µg/ml streptomycin. Mixed glial cell cultures grew at 37 °C in a 5% CO_2_ humidified atmosphere. When confluency was reached, two weeks later, microglial cells were isolated using an anti-CD11b antibody and a magnetic cell sorting (MACS) method following the manufacturer’s instructions (Miltenyi Biotec, The Netherlands). Microglial cells were then plated in culture medium composed of mixed glial cell culture-conditioned medium and DMEM (50/50, v/v). Twenty-four hours after seeding, microglial cultures were treated with 5 µM of A53T α‑synuclein, 20 µM of apomorphine enantiomers (Sigma) or with 5 nM trigonelline (Sigma). In addition, a siRNA-mediated gene silencing approach has also been established on these primary cultures (see the RNA silencing section). For control conditions, we treated the cells using the same volume of vehicle (sterile water) as used in the A53T condition.

The cellular viability was evaluated after each treatment using the MTT method (ability of mitochondrial dehydrogenases from healthy cells to convert MTT (3-[4,5-dimethylthiazol-2-yl]-2,5-diphenyl-tetrazolium bromide) to coloured insoluble formazan crystals) and the LDH cytotoxicity assay [measuring the lactate dehydrogenase (LDH) activity in the supernatant upon release by damaged cells] (Hoenen et al. 2016).

### Phagocytosis Assay

Primary microglial cells were seeded at a density of 2.5 × 10^5^ cells/well into 48-well plates. Cells were incubated for 24 h with the different compounds. Briefly, yellow-green carboxylate-modified FluoSpheres® (1 µm, Thermo Fisher Scientific, Belgium) were resuspended in 25 mM Na_2_HPO_4_, pH 6.0, buffer containing 3% (w/v) bovine serum albumin and water-bath sonicated for 15 min at 25 °C. At the end of the treatment time, the sonicated suspension (2 × 10^9^ microspheres in 12.5 µl) was added to the microglia cultures. The incubation was maintained at 37 °C for 75 min. After three PBS washing steps, a Trypan blue solution (0.1% final) was added in each well in order to quench the extracellular fluorescence (non-phagocytized beads). Fluorescence was measured in a FLUOstar OPTIMA microplate reader (BMG Labtech, Offenburg, Germany) at 520 nm under excitation at 485 nm.

### Immunocytochemistry

Poly-L-lysine coated coverslips were used when seeding microglia at a density of 3 × 10^5^ cells/well into 24-well plates (Hoenen et al. 2016). After 6 h of treatments, cells were successively fixed for 20 min at room temperature with 4% paraformaldehyde (PFA), permeabilized for 5 min in 0.3% Triton X100 in PBS and washed three times with PBS. For the blocking step, cells were incubated for 30 min with 3% (w/v) BSA at room temperature. Microglial cells were stained overnight at 4 °C using a rabbit anti-IBA1 antibody (1:300, Biocare Medical, USA), a rat anti-F4/80 antibody (1:300, Bio-Rad, Belgium), or a rabbit anti-NRF2 antibody (1:300, Invitrogen, Belgium). The next day, cells were incubated with an Alexa Fluor® 488-conjugated anti-rabbit secondary antibody (1:1000, Thermo Fisher Scientific, Belgium), an Alexa Fluor® 488-conjugated anti-rat secondary antibody (1:1000, Thermo Fisher Scientific, Belgium), or an Alexa Fluor® 568-conjugated anti-rabbit secondary antibody (1:1000, Thermo Fisher Scientific, Belgium) for one hour at room temperature. Finally, the glass slides containing the cells were washed in PBS, and were mounted with DAPI Fluoromount-G (SouthernBiotech, USA). Pictures were acquired using an LSM 510 META inverted confocal microscope at a 40 × magnification (Carl Zeiss Micro Imaging, Göttingen, Germany). The open source image-processing software package Fiji® was used to establish microglial cell areas (µm^2^). Regarding the detection of NRF2 in nuclei, pictures were acquired at a 60 × magnification using an Andor Revolution W1 Spinning Disk Confocal system, mounted on a Nikon Ti microscope using Nikon Imaging Software (NIS).

### RNA Silencing

For the silencing of *Nfe2l2*, we used *Nfe2l2*-targeted siRNA. Primary microglial cells were seeded at a density of 4 × 10^5^ cells/well into 24-well plates. Cells were transfected 24 h later with 30 nM *Nfe2l2* small interfering RNA (siRNA) or negative control siRNA (Eurogentec, Belgium) using Lipofectamine® RNAiMAX reagent (Life Technologies, Belgium).

The synthetic *Nfe2l2* siRNAs duplexes are 5′-GACAAGAGCAACUCCAGAAdTdT-3′ and 5′-UUCUGGAGUUGCUCUUGUCdTdT-3′. Following the manufacturer’s instructions, siRNAs and Lipofectamine RNAiMAX were prepared in DMEM, premixed for 5 min and applied to the cells. Following 24 h of transfection, cells were then treated for 6 h with A53T with or without apomorphine enantiomers and harvested for total RNA extraction followed by gene expression analyses.

### RNA Extraction, Reverse Transcription and PCR

Primary microglial cells were seeded at a density of 6 × 10^5^ cells/well into 12-well plates. Total RNA was extracted after treatments using the innuPREP RNA Kits (Westburg, The Netherlands) following the manufacturer’s instructions. Reverse transcription was made with the ImProm-II Reverse Transcription System kit (Promega, The Netherlands). For PCR analyses, iQ™ SYBR Green Supermix (Promega) and synthesized complementary DNA (cDNA) were used on a Bio-Rad iCycler (iQ5 Real-Time PCR Detection System, Bio-Rad). Primer sequences are summarized in Table 1. Gene expression was calculated with the comparative threshold cycle (*C*_t_) method using the 2^−ddCt^ formula, with ddCt = (*C*_t, target_ − *C*_t, *Rpl27*_)_treated sample_ − (*C*_t, target_ − *C*_t, *Rpl27*_)_control sample_. To normalize the target genes, *Rpl27* was used as the housekeeping gene (coding for a ribosomal protein).

**Table 1:** Sequences of the different real-time PCR primers

Target gene (Accession number)	Forward	Reverse
*Cxcl10* (NM_021274)	TTCTGCCTCATCCTGCTG	AGACATCTCTGCTCATCATTC
*Drd1* (NM_010076)	CAGACACAAATAAACACAAGGTA	AACATTCAGAACACAATAGTAGC
*Drd2* (NM_010077)	ACTCAGATGCTTGCCATTG	GGATGTGCGTGATGAAGAA
*Drd3* (NM_007877)	CTACGCCCTGTCCTACTGT	CCACCTGTCACCTCCAAG
*Drd4* (NM_007878)	GTGTTGGACGCCTTTCTTCG	GGGTTGAGGGCACTGTTGA
*Drd5* (NM_013503)	CTGCGAGCATCCATCAAG	CACAAGGGAAGCCAGTCC
*Hmox1* (NM_010442)	TCAGAAGGGTCAGGTGTC	TCAGGGAAGTAGAGTGGG
*Hmox2* (NM_001136066)	CACAAACTACTCAGCCAC	CTTGGTCCCTTCCTTCAG
*Nfe2l2* (NM_010902)	TGAAGCTCAGCTCGCATTGA	TGGGCGGCGACTTTATTCTT
*Nos2* (NM_010927)	AGCCCTCACCTACTTCCTG	CAATCTCTGCCTATCCGTCTC
*Nqo1* (NM_008706)	GGTAGCGGCTCCATGTACTC	CATCCTTCCAGGATCTGCAT
*Ptgs2* (NM_011198)	GCCTGGTCTGATGATGTATGC	GAGTATGAGTCTGCTGGTTTGG
*Rpl27* (NM_011289)	ACATTGACGATGGCACCTC	GCTTGGCGATCTTCTTCTTG
*Tnf* (NM_013693)	GGTTCTGTCCCTTTCACTCAC	TGCCTCTTCTGCCAGTTCC

All the mouse primer sequences (5′-3′) have been generated using the Primer3/BLAST tool

### Quantification of Pro-Inflammatory Mediators

Supernatants were collected after treatments performed in 12-well plates (6 × 10^5^ cells/well) for 6 h. The production of prostaglandins E_2_ (PGE_2_), CXCL10 and TNFα was quantified with an Enzyme ImmunoAssay kit (Assay Designs, USA) or a sandwich Enzyme Linked ImmunoSorbent Assay (R&D Systems, USA). A microplate reader (TECAN) was used to measure the absorbance at 540 nm and a 4-parameter logistic (4PL) standard curve fit led us to calculate protein concentrations.

### Western Blot

Following 3 h of treatments, total proteins were extracted from 6-well plates (1 × 10^6^ cells/well) with RIPA buffer containing 1% phosphatase/protease inhibitor cocktail (Pierce). Nuclear and cytoplasmic proteins were extracted from 25 cm^2^ flasks (density of 4 × 10^6^ cells/flask), after 2 h of treatments, using the nuclear extract kit (Active Motif, CA). Protein levels were determined by the Bio-Rad Protein Assay Kit.

Twenty µg (total and cytoplasmic proteins) or 10 µg (nuclear proteins) were loaded on a 12% SDS-PAGE gel and transferred to a nitrocellulose membrane (Bio-Rad). Membranes were incubated with either anti-ERK (rabbit, 1:20,000 dilution, ref. M5670, Sigma), anti-phospho-ERK (rabbit, 1:2000 dilution, ref. 4370, Bioke), anti-p38 (rabbit, 1:1000 dilution, ref. 506,123, Calbiochem), anti-phospho-p38 (rabbit, 1:1000 dilution, ref. 4511, Bioke), anti-STAT1 (mouse, 1:500 dilution, ref. BD610116, BD Biosciences), anti-phospho-STAT1 (rabbit, 1:1000 dilution, ref. 7649, Bioke), anti-α-tubulin (mouse, 1:5000 dilution, ref. ab7291, AbCam), anti-NRF2 (rabbit, 1:500 dilution, ref. PA5-27,882, Invitrogen) or anti-HDAC1 (mouse, 1:2000 dilution, ref. ab46985, AbCam) antibody. After washing steps, membranes were incubated with an anti-mouse or anti-rabbit IgG-HRP antibody (1:2000 dilution, ref. NXA931 and NA934, Amersham Biosciences). Proteins of interest were revealed using SuperSignal West Femto Maximum Sensitivity Substrate (Pierce) on a ChemiDoc™ XRS + System (Bio-Rad).

### Statistical Analysis

GraphPad Prism 8.0 (GraphPad Software, Inc., San Diego, CA) was used to assess significant differences. Results are shown as mean ± standard error of the mean (SEM) from a minimum of three independent experiments. Differences between treatments were analysed by a one-way analysis of variance (ANOVA) followed by a Tukey’s multiple comparisons test. When normality could not be established, different group mean values were compared using a non-parametric analysis of variance (Kruskal–Wallis test) followed by a Dunn’s post-hoc test. For real-time PCR data, the statistical analysis of gene expression was performed on the delta-Ct values (Ct_gene of interest_ − Ct_housekeeping gene_). The analyses of protein release quantification data as well as the quantification of the different proteins detected by western-blot (ratios) were performed on log-transformed data. Differences between groups were considered as significant when *p* values were less than 0.05.

## Results

### Apomorphine Co-Treatment Decreases A53T-Induced Microglial Reactivity and Modulates Phagocytosis Capacity

Primary microglia were exposed to the A53T mutant α-synuclein protein. We first confirmed that our α-synuclein preparation was free of endotoxin contamination (LAL assays; data not shown). Furthermore, MTT and LDH assays showed no viability changes following exposure of our cultures to α-synuclein, apomorphine, trigonelline and siRNAs (data not shown).

**Fig. 1:**
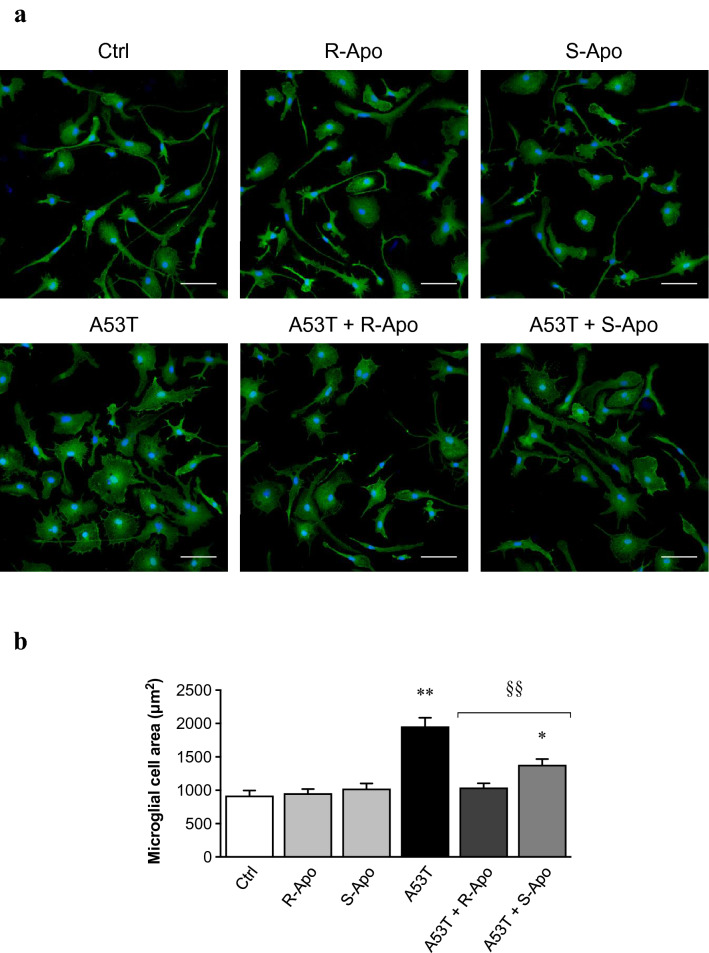
Apomorphine enantiomers prevent changes in microglial cell morphology induced by A53T exposure. Primary microglia were co-treated for 6 h with the mutant A53T α-synuclein protein (5 µM) as well as with 20 µM of R-apomorphine (R-Apo) or S-apomorphine (S-Apo) (**a**). Cells were labelled with the microglial marker IBA1 (green) and counterstained with nuclear stain DAPI (blue). Scale bar: 25 µm. To establish morphological changes, microglial cell areas were determined after treatments (mean ± SEM of 20 measures per condition) (**b**). *p < 0.05, **p < 0.01, significantly different from control condition (untreated cells); §§ *p* < 0.01, significantly different from A53T-treated cells

Upon A53T exposure, microglial morphology changed considerably as observed by immunocytochemistry (Fig. 1a). Indeed, the area covered by microglial cells (Fig. 1b) was strongly increased (2.1-fold compared to control condition). When co-treated with R-apomorphine and A53T protein, normal cell morphology was restored. In this condition, the cell area was similar to the control condition (Fig. 1b). S-apomorphine co-treatment partially reduced the increase of cell area induced by the presence of the A53T protein (− 70% compared to the A53T condition). It should be noted that no change of morphology was observed after single treatment with each apomorphine enantiomer. Furthermore, no significant change in cell proliferation was observed in all conditions (data not shown).

**Fig. 2:**
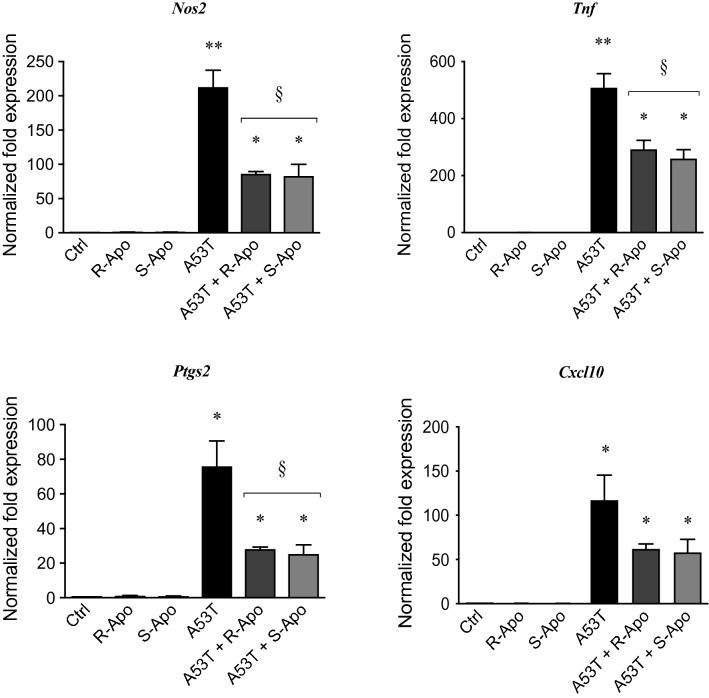
Apomorphine enantiomers down-regulate A53T-induced pro-inflammatory gene overexpression. Following exposure of primary mouse microglia to A53T protein (5 µM) and R- or S-apomorphine (20 µM) for 6 h, expressions of pro-inflammatory genes (*Cxcl10*, *Nos2*, *Ptgs2* and *Tnf*) were analysed by real-time PCR. Gene expressions were normalized to *Rpl27* expression level and control levels were fixed at 1.0. Results are given as mean ± SEM (*n* = 4 independent experiments). *p < 0.05, **p < 0.01, significantly different from control condition (untreated cells); § *p* < 0.05, significantly different from A53T-treated cells

In addition to these morphological changes, we also assessed the treatment effects on the expression of pro-inflammatory genes in microglial cells by real-time PCR (Fig. 2). Transcription levels of *Cxcl10*, *Nos2*, *Ptgs2* and *Tnf* were strongly increased 6 h after the treatment with A53T. Apomorphine co-treatments were able to significantly decrease the A53T-induced up-regulation of these pro-inflammatory genes by 60% for *Nos2*, 45% for *Tnf* and 65% for *Ptgs2*, whereas for *Cxcl10* expression, the decrease was not significant (*p* = 0.08). Interestingly, the downregulation of these pro-inflammatory gene expression levels appeared to be enantiomer-independent. Finally, both apomorphine enantiomers alone had no effect on the expression of these pro-inflammatory genes.

**Fig. 3:**
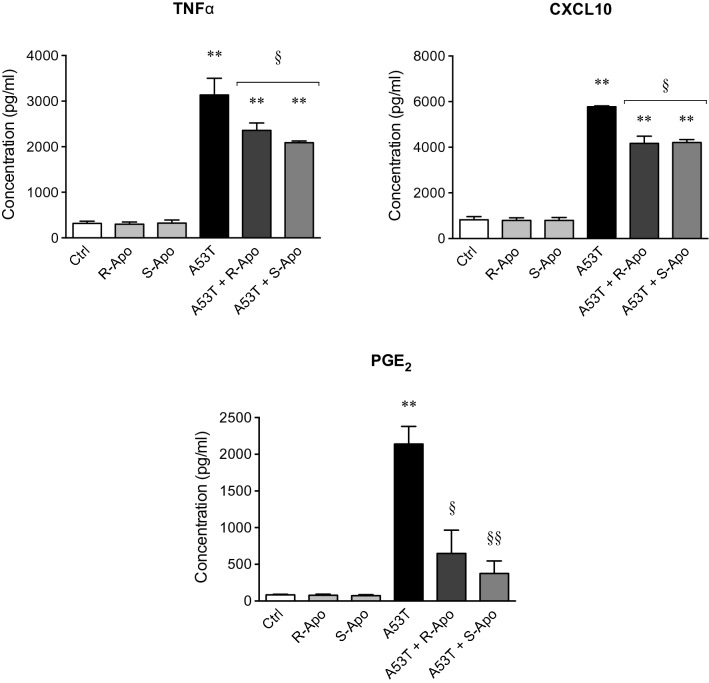
Apomorphine enantiomers decrease the A53T-induced release of pro-inflammatory mediators. CXCL10, PGE_2_ and TNFα protein releases were quantified in the supernatant of microglial cultures after treatments with A53T protein (5 µM) and R- or S-apomorphine (20 µM) for 6 h. Results are given as mean ± SEM (*n* = 4 independent experiments). **p < 0.01, significantly different from control condition (untreated cells); § *p* < 0.05, §§ *p* < 0.01, significantly different from A53T-treated cells

As a result of *Cxcl10*, *Ptgs2* and *Tnf* gene upregulation, the release of CXCL10, PGE_2_ and TNFα increased significantly 6 h after A53T treatment (Fig. 3). Co-treatments with apomorphine enantiomers partially reduced the production of these pro-inflammatory mediators in an enantiomer-independent manner. Thus, R/S-apomorphine reduced the A53T-induced TNFα, CXCL10 and PGE_2_ releases by 35%, 30% and 75%, respectively. Single treatments with apomorphine enantiomers did not affect the production of these pro-inflammatory mediators.

**Fig. 4:**
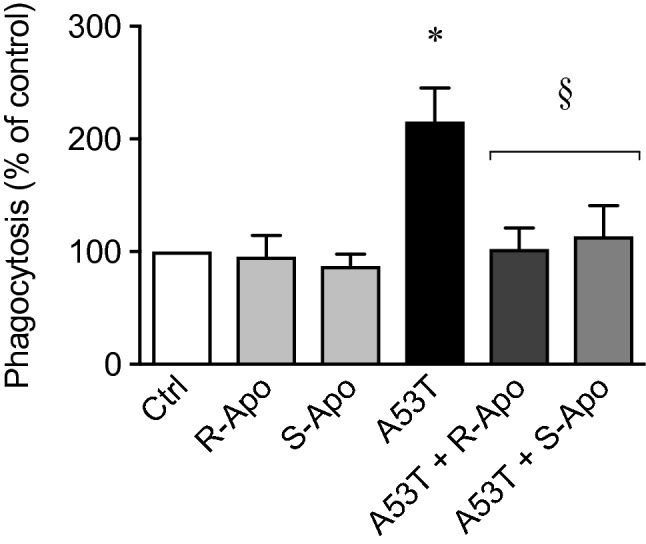
Microglial phagocytosis capacity is increased upon A53T exposure but can be modulated by apomorphine. Phagocytosis capacity was measured after incubation of primary microglia with the A53T mutant protein (5 µM) with or without apomorphine enantiomers co-treatments (20 µM) for 24 h. Results are given as mean ± SEM (*n* = 3 independent experiments). *p < 0.05, significantly different from control condition (untreated cells); § *p* < 0.05, significantly different from A53T-treated cells

After 24 h of microglial exposure to A53T protein, phagocytosis activity was increased by 2.1-fold (Fig. 4). Co-treatments with both apomorphine enantiomers totally inhibited the increase in phagocytosis.

### JAK-STAT and MAPK Signalling Pathways are Regulated by R/S-Apomorphine Enantiomers in A53T-Activated Microglial Cells

**Fig. 5:**
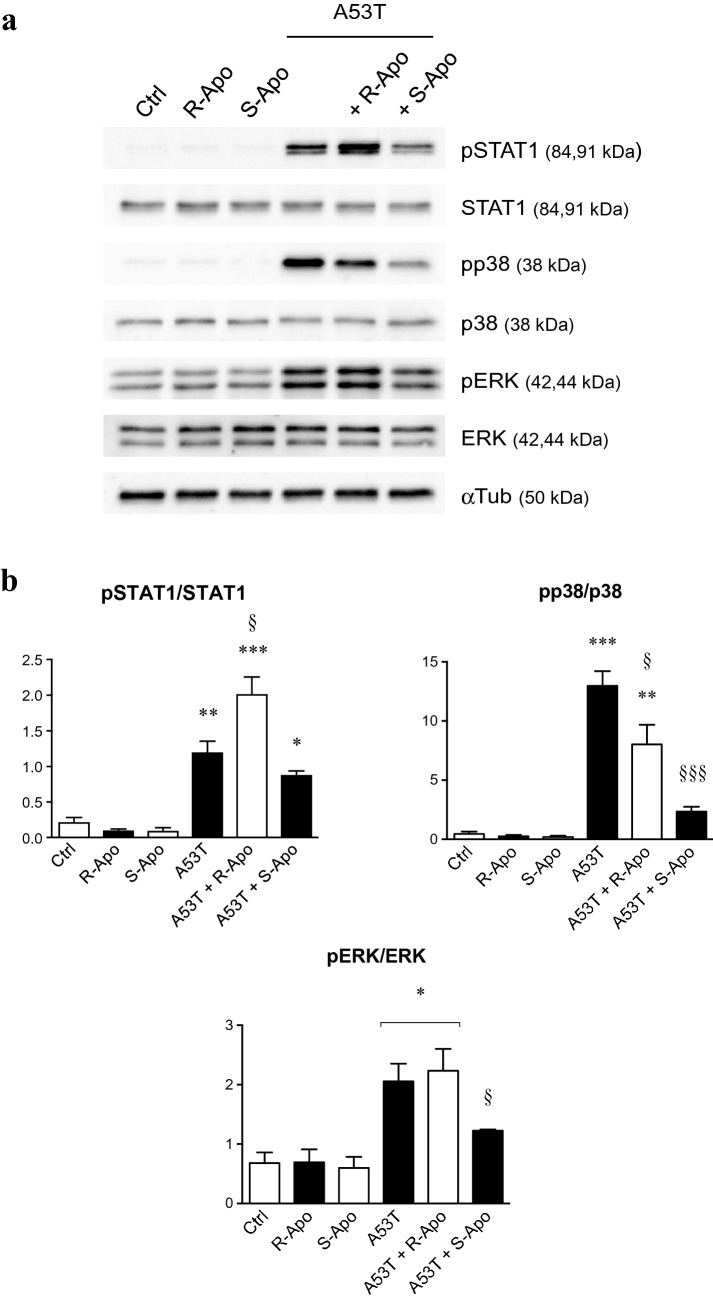
A53T-induced STAT1 phosphorylation and MAPKs activation are modulated following apomorphine exposure. After 3 h of co-treatments with A53T protein (5 µM) and apomorphine enantiomers (20 µM), microglia lysates were subjected to western-blot analysis (**a**) to determine the expression of phosphorylated forms of ERK (pERK), p38 (pp38) and STAT1 (pSTAT1). α-Tubulin (αTub) was used as a reference protein. Phosphorylated versus non-phosphorylated proteins ratios, called pERK/ERK, pp38/p38 and pSTAT1/STAT1, are shown in **b**. Results are given as mean ± SEM of at least three independent experiments. *p < 0.05, **p < 0.01, ***p < 0.005, significantly different from control condition (untreated cells); § *p* < 0.05, §§§ *p* < 0.005 significantly different from A53T-treated cells

To decipher the involved mechanisms in our study, total proteins were extracted after 3 h of A53T exposure. In this condition, phosphor-STAT1 (pSTAT1), phospho-p38 (pp38) and phospho-ERK (pERK) expression were significantly increased compared to the control condition (Fig. 5a, b). Concomitantly, levels of non-phosphorylated STAT1, p38 and ERK protein expression remained unchanged. Compared to A53T treatment alone, the addition of R-apomorphine increased the phosphorylation of STAT1 (+ 68%) but decreased the phosphorylation of p38 (− 38%) whereas pERK expression remained unchanged. Furthermore, co-treatment with S-apomorphine strongly decreased the phosphorylation of STAT1, p38 and ERK by 26%, 80% and 40%, respectively.

Of note, A53T exposure alone or in combination with apomorphine enantiomers was not able to modulate JNK and phospho-JNK MAPK expression in microglia (data not shown).

### R/S-Apomorphine Treatments Increase the Nuclear Recruitment of NRF2 and Expression of NRF2-Target Genes (*Hmox1*, *Nqo1*)

**Fig. 6:**
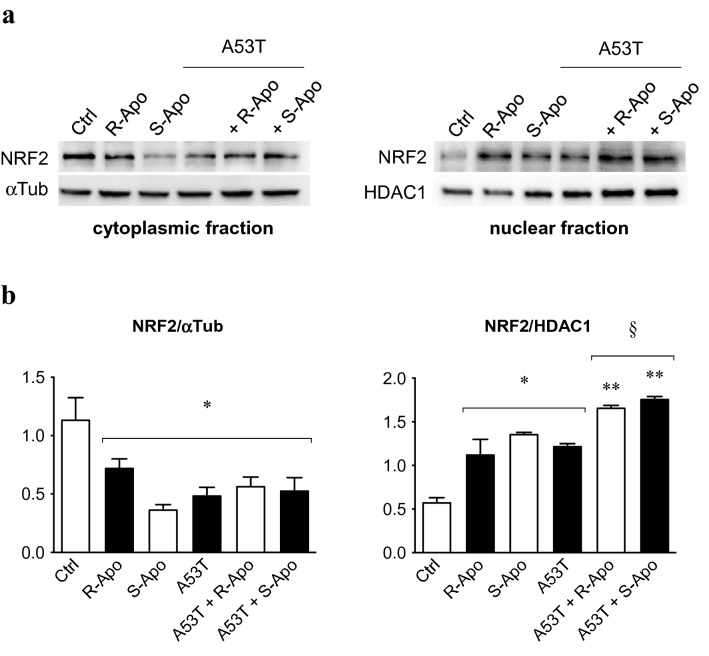
Apomorphine enantiomers induce nuclear recruitment of NRF2. The localization of NRF2 protein was evaluated after a 5 µM A53T and 20 µM apomorphine enantiomers exposure for 2 h on microglial cells (**a**). Cytoplasmic and nuclear NRF2 protein expression (80 kDa) was evaluated in our experimental conditions. α-Tubulin (αTub, 50 kDa) and histone deacetylase 1 (HDAC1, 55 kDa) were used as reference proteins. Detected proteins were then quantified and normalized (**b**). Results are given as mean ± SEM of at least three independent experiments. *p < 0.05, **p < 0.01, significantly different from control condition (untreated cells); § *p* < 0.05 significantly different from A53T-treated cells

**Fig. 7:**
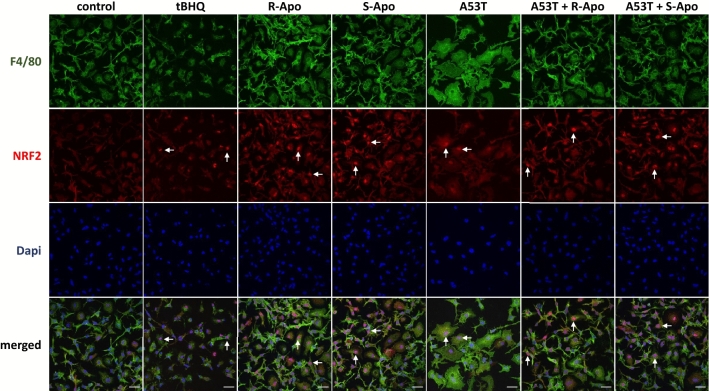
NRF2 transcription factor translocates to the nucleus upon apomorphine exposure. Primary microglia were co-treated for 6 h with the mutant A53T α-synuclein protein (5 µM) as well as with 20 µM of R-apomorphine (R-Apo) or S-apomorphine (S-Apo). Tert-butylhydroquinone (tBHQ) was used as a positive control for NRF2 nuclear translocation. Cells were labelled for the microglial marker F4/80 (green) or NRF2 (red) and counterstained with nuclear stain DAPI (blue). White arrows point to nuclear localization of NRF2. Scale bar: 25 µm

We determined NRF2 protein levels in both cytoplasmic and nuclear fractions of microglial cells (Fig. 6a, b). These results highlighted a decrease of cytoplasmic NRF2 protein upon A53T exposure (− 55%) as well as with both apomorphine enantiomers alone by − 35% for the R-Apo and − 65% for the S-Apo, respectively. Furthermore, co-treatments with A53T and apomorphine enantiomers reduced the cytoplasmic NRF2 level by 50% as compared to the control. As a result of a decrease of cytoplasmic NRF2, we observed an increase of nuclear NRF2 recruitment in all experimental conditions. Indeed, the nuclear NRF2 translocation was increased upon A53T exposure (2.3-fold) as well as upon R-Apo (2.2-fold) and S-Apo (2.5-fold) exposure. Co-treatments with A53T and R-Apo or S-Apo induced a higher NRF2 recruitment, which appeared to be enantiomer independent (approximatively 3.5-fold stronger compared to control condition and 1.6-fold stronger compared to A53T exposure alone). The nuclear localization of NRF2 was also evaluated by immunofluorescence (Fig. 7). Tert-butylhydroquinone (tBHQ) was used as a positive control to induce the nuclear translocation of NRF2. Unlike the control condition, all treatments increased the fluorescence intensity in the nucleus (white arrows) highlighting the translocation of NRF2 from the cytoplasm to the nucleus. These data confirm our western-blot results (Fig. 6).

**Fig. 8:**
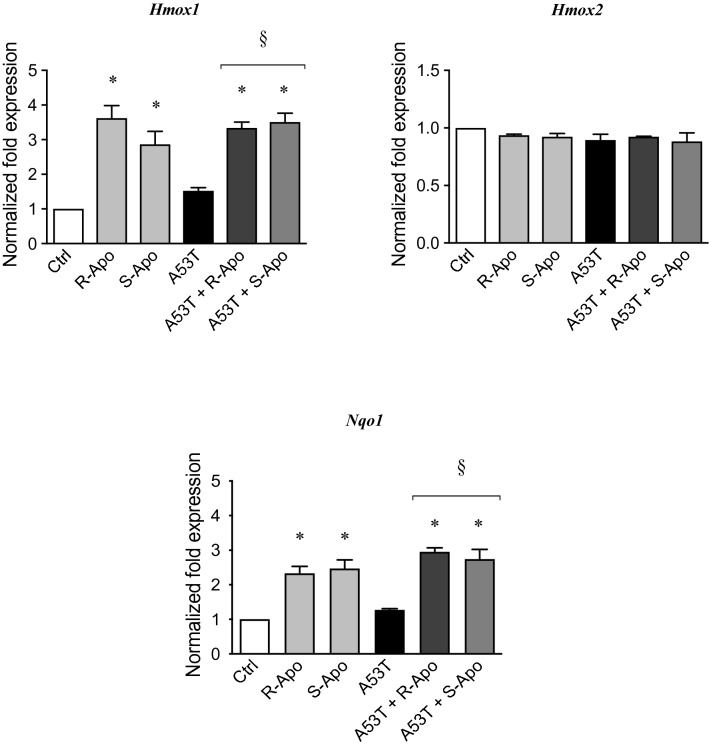
Apomorphine enantiomers upregulate NRF2-target genes. Following exposure of primary mouse microglial cells to A53T protein (5 µM) and apomorphine enantiomers (20 µM) for 6 h, heme oxygenase-1 (*Hmox1*), heme oxygenase-2 (*Hmox2*), and NAD(P)H Quinone Oxidoreductase 1 (*Nqo1*) expressions were analysed by real-time PCR. Gene expressions were normalized to *Rpl27* expression level and control levels were fixed at 1.0. Results are given as mean ± SEM of at least three independent experiments. *p < 0.05, significantly different from control condition (untreated cells); §*p* < 0.05 significantly different from A53T-treated cells

In order to validate the recruitment of NRF2 in our conditions, we assessed the gene expression of Heme Oxygenases (*Hmox1* and *Hmox2*) and NAD(P)H quinone oxidoreductase 1 (*Nqo1*) by real-time PCR (Fig. 8). In contrast to *Hmox2*, *Hmox1* and *Nqo1* are both considered as NRF2-target genes. While treatment by either R- or S-Apo similarly increased the expression of *Hmox1* and *Nqo1*, A53T exposure did not significantly increase their expression. Furthermore, co-treatments with A53T and R-Apo or S-Apo did not differ regarding *Hmox1* and *Nqo1* overexpression in comparison to the effects of R-Apo or S-Apo alone. The expression of *Hmox2* did not change between all treatments.

### Inhibition of the NRF2-ARE Pathway Abrogates the Beneficial Role of Apomorphine Enantiomers

**Fig. 9:**
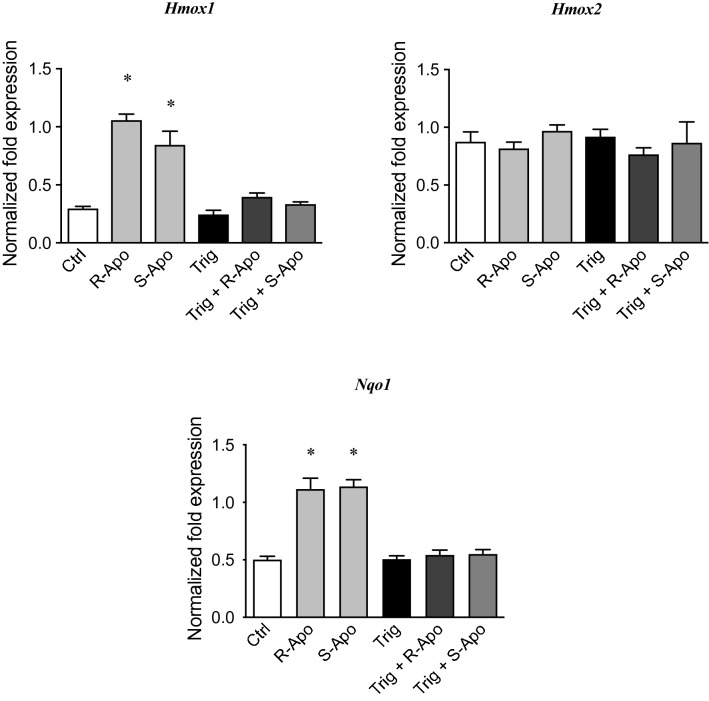
Trigonelline exposure inhibits the recruitment of NRF2 transcription factor. Primary microglial cultures were pretreated for 1 h with 5 nM trigonelline. Cells were then treated for 6 h with apomorphine enantiomers (20 µM). Heme oxygenase-1 (*Hmox1*), heme oxygenase-2 (*Hmox2*), and NAD(P)H Quinone Oxidoreductase 1 (*Nqo1*) expressions were analysed by real-time PCR. Gene expressions were normalized to *Rpl27* expression level. Results are given as mean ± SEM (*n* = 4 independent experiments). *p < 0.05, significantly different from control condition (untreated cells)

**Fig. 10:**
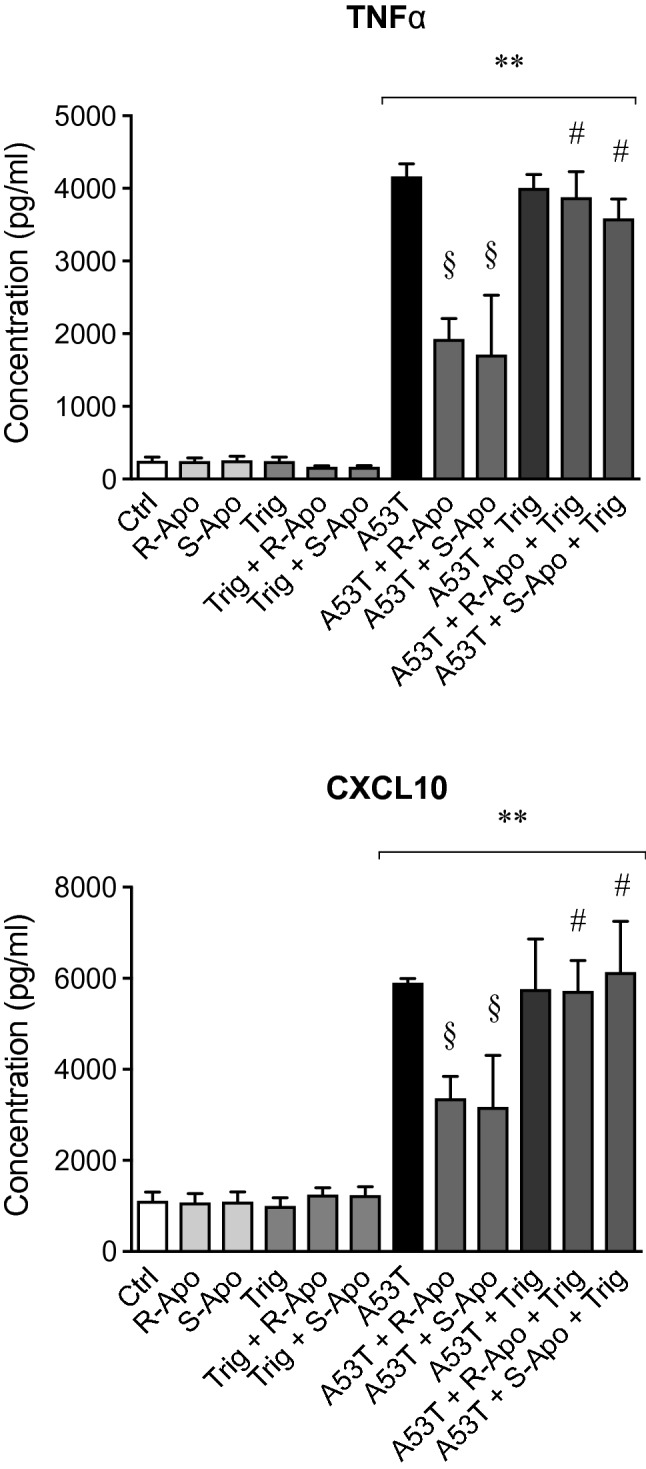
Trigonelline exposure abolishes the effects of apomorphine enantiomers on microglial reactivity. CXCL10 and TNFα protein releases were quantified by ELISA in the supernatant of microglial cultures after 1 h of pre-treatment with 5 nM trigonelline followed by 6 h of co-treatments with the A53T protein (5 µM) and apomorphine enantiomers (20 µM). Results are given as mean ± SEM (*n* = 4 independent experiments). **p < 0.01, significantly different from control condition (untreated cells); § *p* < 0.05, significantly different from A53T-treated cells; # *p* < 0.05, significantly different from the same treatment without trigonelline

**Fig. 11:**
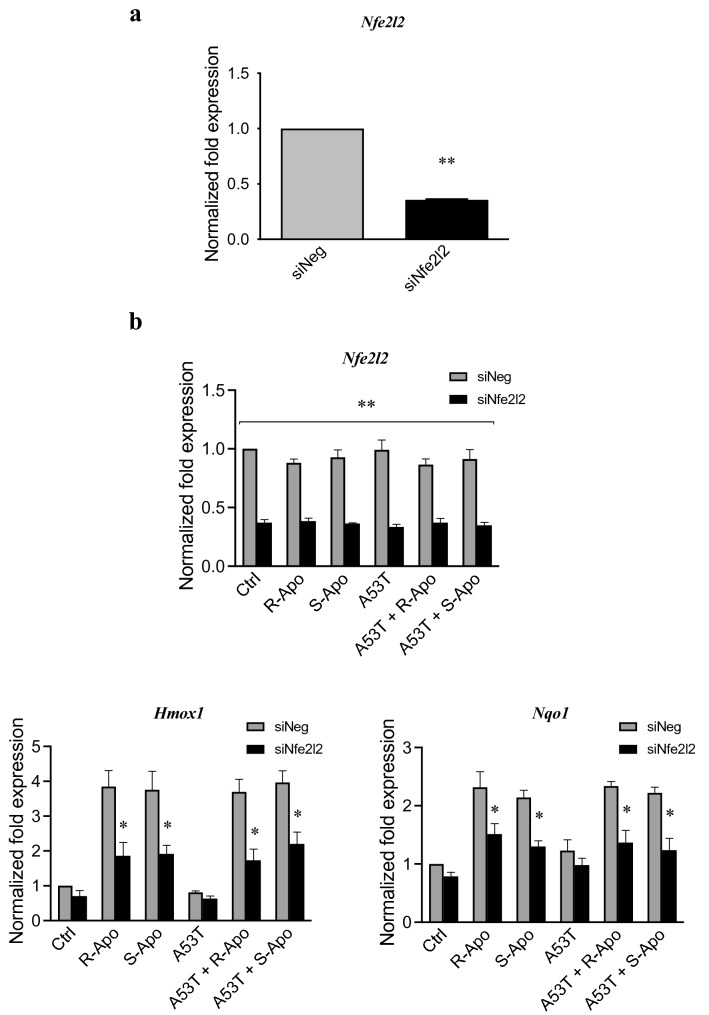
*Nfe2l2* silencing inhibits apomorphine-induced overexpression of NRF2 target genes. To silence *Nfe2l2* expression, cells were transfected with 30 nM *Nfe2l2* siRNA (siNfe2l2) or negative control siRNA (siNeg) using Lipofectamine® RNAiMAX reagent. After 24 h of transfection, *Nfe2l2* gene expression was analysed by real-time PCR (**a**). Following siRNA transfection, cells were co-treated for 6 h with the A53T protein (5 µM) and apomorphine enantiomers (20 µM). *Nfe2l2*, *Hmox1*, and *Nqo1* expression were analysed by real-time PCR (**b**). Gene expression was normalized to *Rpl27* expression level and control levels (siNeg condition) were fixed at 1.0. Results are given as mean ± SEM (*n* = 4 independent experiments). *p < 0.05, **p < 0.01, significantly different from siNeg condition (with the same treatment)

**Fig. 12:**
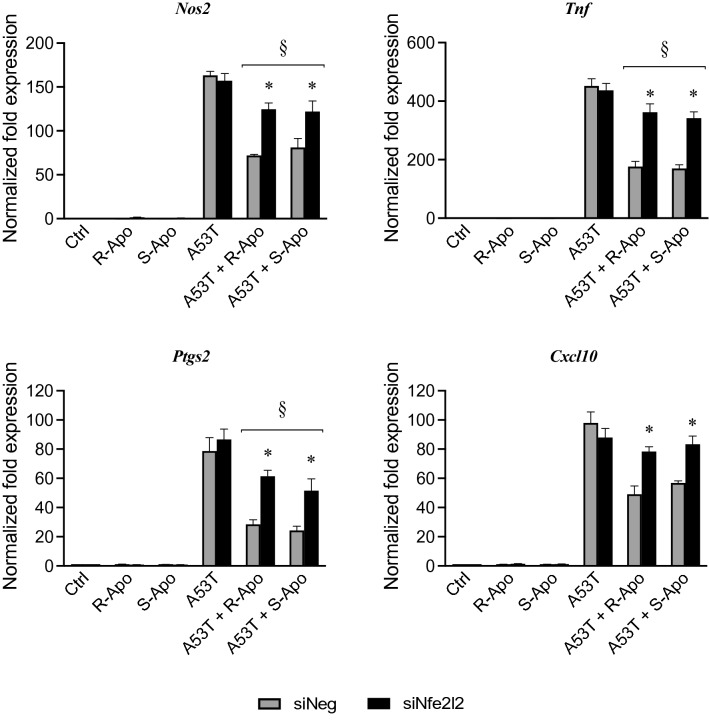
*Nfe2l2* silencing reverses the anti-inflammatory properties of apomorphine. Following *Nfe2l2* inhibition, primary mouse microglia were exposed to A53T protein (5 µM) and R- or S-apomorphine (20 µM) for 6 h. Expressions of pro-inflammatory genes (*Cxcl10*, *Nos2*, *Ptgs2* and *Tnf*) were analysed by real-time PCR. Gene expressions were normalized to *Rpl27* expression level and control levels were fixed at 1.0. Results are given as mean ± SEM (*n* = 4 independent experiments). *p < 0.05, significantly different from siNeg condition (with the same treatment); § *p* < 0.05, significantly different from A53T-treated cells

The in vitro effects of a pharmacological inhibition and genetic silencing of NRF2 were examined in our primary microglial cultures. Trigonelline, a plant alkaloid, was used as an effective inhibitor of NRF2 recruitment. Pre-treating with trigonelline (5 nM) during 1 h totally prevented the R/S-apomorphine induced overexpression of the NRF2-target genes, *Hmox1* and *Nqo1* (Fig. 9). These treatments did not change the expression of *Hmox2*. Trigonelline exposure was also able to evenly block the anti-inflammatory effects of R-Apo or S-Apo. Indeed, pre-treating with trigonelline in addition to A53T and apomorphine restored TNFα and CXCL10 to the same protein levels as upon A53T exposure alone (Fig. 10). In these conditions, we obtained similar results for *Tnf* and *Cxcl10* gene expression assessed by real-time PCR (data not shown). To further demonstrate the contribution of the NRF2 signaling pathway, *Nfe2l2* gene knockdown model was established using *Nfe2l2* siRNA transfection. Thus, after 24 h of transfection, *Nfe2l2* mRNA expression was strongly decreased by − 65% (Fig. 11a). Furthermore, the results indicated that *Nfe2l2* knockdown largely inhibited the expression of *Hmox1* and *Nqo1* (Fig. 11b) and strongly decreased the anti-inflammatory effects of R- and S-apomorphine as also observed by real-time PCR for *Cxcl10*, *Nos2*, *Ptgs2* and *Tnf* expression (Fig. 12).

## Discussion

The term “neuroinflammation” broadly defines the inflammatory processes occurring in the central nervous system where glial cells, in particular astrocytes and microglia, are recognized as principal players. Neuroinflammation, neuronal dysfunction or neuronal death leading to neurodegeneration are linked to an increase of potentially neurotoxic molecules like pro-inflammatory cytokines/chemokines, ROS and RNS. The brain resident innate immune cells, microglia, are constantly monitoring the brain. A short-term microglial activation is believed to be neuroprotective (Ekdahl et al. 2009), while a prolonged microglial activation can further increase tissue damage and negatively impact disease outcome (Block et al. 2007; Block and Hong 2007; Lull and Block 2010) and, in case of neurodegenerative diseases, contribute to neuronal depletion.

Characterized by the deposition of misfolded protein aggregates, predominantly composed of α-synuclein, and the depletion of striatal dopamine following the progressive degeneration of dopaminergic neurons in the Substantia Nigra pars compacta (SNpc), Parkinson’s disease is the most common neurodegenerative movement disorder. Most PD cases are sporadic but inherited PD forms can be caused by point mutations of the gene encoding α-synuclein protein. In a previous work, we described that the microglia activation level was α-synuclein protein dependent (Hoenen et al. 2016). Indeed, wild-type α-synuclein (WT α-syn) and the three corresponding mutants (A30P, A53T and E46K) differentially activate primary microglia. Exposure to A53T protein was able to activate primary murine microglial cultures more strongly through the recruitment of transcription factors such as AP-1, NFĸB, and NRF2.

In our primary murine microglial culture, we have highlighted the expression of dopamine receptors (Table 2). According to their downstream signalling pathways, these G-protein coupled receptors are classified in two families called D1-like (D1, D5) and D2-like receptors (D2, D3 and D4). Both families have opposite effects: D1-like receptors promote the production of cyclic adenosine monophosphate (cAMP), while D2-like receptors family suppresses cAMP production (Missale et al. 1998; Gurevich et al. 2016; Xia et al. 2019). Considering the Brain RNA-Seq data from Barres’ Lab (www.brainrnaseq.org), the expression levels of the different dopamine receptors (*Drd1*–*Drd5*) are much lower in microglia than in neuronal cells (Wei et al. 2018; Mishra et al. 2018). Depending on the agonists/antagonists used, recent works showed that modulating dopamine receptors could reduce or even prevent microglial polarization towards a pro-inflammatory state (Huck et al. 2015; Kalkman and Feuerbach 2016; Dominguez-Meijide et al. 2017; Fan et al. 2018; Singh et al. 2018). Targeting dopaminergic receptors might provide an opportunity to control neuroinflammation thereby improving some CNS diseases. Commonly used as an antiparkinsonian drug, apomorphine has been described to be the most effective dopamine agonist when subcutaneously administrated (Carbone et al. 2019). This chiral molecule is a non-selective dopamine receptor agonist, which activates both D1/D2 receptor families. Contrary to the R-apomorphine, the S-isomer lacks dopamine receptor agonist activity (Li et al. 2006).

**Table 2:** Threshold cycle (*C*_t_) values of the dopamine receptor genes in primary microglia

Target gene (Accession number)	Primary microglia (*C*_t_) (mean ± SEM)
*Drd1* (NM_010076)	31.5 ± 0.5
*Drd2* (NM_010077)	36.0 ± 0.5
*Drd3* (NM_007877)	35.8 ± 0.6
*Drd4* (NM_007878)	32.0 ± 0.5
*Drd5* (NM_013503)	31.4 ± 0.5
*Rpl27* (NM_011289)	17.5 ± 0.2

*C*_t_ values were determined in control condition (untreated cells) in primary microglia. Results are shown as mean ± SEM (*n* = 5 independent experiments)

Our in vitro data showed that apomorphine co-treatments decreased the A53T-induced microglial reactivity. A change in morphology is a known hallmark of microglial activation. Apomorphine co-treatments totally (R-Apo) or partially (S-Apo) prevented the increase of the microglial cell area observed upon A53T exposure (Fig. 1). Both enantiomers strongly decreased the overexpression (Fig. 2) of pro-inflammatory markers (*Nos2*, *Tnf*, *Ptgs2*, *Cxcl10*) as well as the release of the corresponding mediators (TNFα, PGE_2_, CXCL10) (Fig. 3) induced by the presence of the A53T mutant. These results are consistent with the observed changes in cell morphology (Fig. 1). Moreover, R-Apo and S-Apo totally restored microglial phagocytosis capacity back to control level (Fig. 4). Thus, both enantiomers were able to decrease A53T effects on primary murine microglia to a similar extent. However, these inhibitory effects appear to be isomer independent. Since S-apomorphine lacks dopamine receptor agonist activity, it is likely that the observed effects of R-Apo are dopamine receptor independent.

The signal transduction was subsequently evaluated in our different experimental conditions. As previously described (Hoenen et al. 2016), an A53T exposure induced the phosphorylation of STAT1, p38 and ERK MAPK in murine microglia (Fig. 5). A co-treatment with S-Apo partially reduced A53T-induced phosphorylation of these three proteins. On the other side, R-Apo/A53T co-treatment reduced more slightly the phosphorylation of p38 MAPK. It should also be noted that this R-Apo/A53T co-treatment did not decrease the recruitment of ERK and even increased the phosphorylation level of STAT1. It has been described that ERK activation can act downstream of STAT signalling during microglial activation (Kim et al. 2002; Tichauer et al. 2007), therefore confirming our findings. Finally, apomorphine enantiomers modulated the MAPK phosphorylation in activated microglia, but not in “resting” microglia (control condition).

In addition to its action on dopaminergic receptors, apomorphine is also a powerful antioxidant and free radical scavenger (Ribarič 2012). These apomorphine effects have been shown in both in vitro and in vivo experiments (Gassen et al. 1996; Grünblatt et al. 1999; Hara et al. 2006; Mead et al. 2013). Several previous studies described that apomorphine, a catechol derivative, is able to activate the Kelch-like ECH-associated protein 1-Nfe2l2-Antioxidant Response Element pathway, commonly called KEAP1-NRF2-ARE pathway, in the mammalian CNS (Hara et al. 2006; Mead et al. 2013). Based on its chemical properties, it has been described that apomorphine autoxidation occurs spontaneously resulting in the formation of quinone derivatives and ROS (Kaul and Brochmann-Hanssen 1961; El-Bachá et al. 2000; Hara et al. 2006). ROS play a critical role in the dissociation of the weak interaction between KEAP1 and NRF2, the two protagonists of the NRF2 signalling pathway (Wakabayashi et al. 2004; Hayes et al. 2010). Detached from KEAP1, NRF2 translocates into the nucleus and transactivates a battery of genes containing ARE sequences (Consensus motif: 5′-TGACnnnGC-3′). This NRF2 transcription factor regulates gene responses to prevent oxidative cell damage, inflammation and tumorigenesis (Johnson et al. 2008; Sivandzade et al. 2019b). Many genes encoding detoxification and antioxidant enzymes have been described to be NRF2-dependent (Ahmed et al. 2017).

We previously described that A53T exposure increases the nuclear translocation of the NFĸB p65 subunit in primary microglia (Hoenen et al. 2016). However, it is assumed that NRF2 negatively regulates NFĸB signalling (Thimmulappa et al. 2006; Bellezza et al. 2012; Liu et al. 2020). Therefore, our results suggest that the NRF2 recruitment inhibits the overexpression of pro-inflammatory genes by interfering with the p65-mediated transcriptional activity (Kobayashi et al. 2016; Kim et al. 2020). Herein, we established a nuclear NRF2 recruitment after 2 h of treatments which can be explained by the dissociation of the NRF2-KEAP1 complex. This is confirmed by an increase of the NRF2 level in the nucleus concomitant to a decrease in the cytoplasmic compartment (Fig. 6a, b). We have thus shown that R- and S-apomorphine treatments induced a NRF2 translocation upon A53T exposure in microglial cells. This is in accordance with the ability of A53T to generate ROS in our microglial cultures (Hoenen et al. 2016). In addition, NRF2 levels in the nucleus were higher upon A53T/apomorphine co-treatments. Immunofluorescent analyses also confirmed the significant increase of NRF2 levels in the nucleus (Fig. 7). To prove the activation of the NRF2 pathway, we focused on its target genes, heme oxygenase-1 (*Hmox1*) and NAD(P)H quinone oxidoreductase 1 (*Nqo1*) (Kansanen et al. 2013; Loboda et al. 2016). After 6 h of treatments, R-Apo and S-Apo alone or in co-treatment with the A53T protein were able to increase *Hmox1* and *Nqo1* expression (Fig. 8). Both apomorphine enantiomers upregulated *Hmox1* and *Nqo1* expression in a same manner. In contrast, another member of the heme oxygenase family, *Hmox2*, a gene not inducible by NRF2, was not sensitive to the different treatments. Even though NRF2 was translocated into the nucleus 2 h after A53T treatment, the expression of *Hmox1* and *Nqo1* did not appear to be significantly upregulated in this condition after 6 h (*p* = 0.078 and 0.091, respectively).

We subsequently wanted to confirm that the activation of NRF2 signalling contributed to the apomorphine effects on microglia. To tackle this question, the use of the NRF2 inhibitor trigonelline (Boettler et al. 2011; Zhou et al. 2012) allowed us to inhibit the nuclear recruitment of this transcription factor (Fig. 9) and to significantly block the anti-inflammatory effects of apomorphine (Fig. 10). Furthermore, the use of specific *Nfe2l2* siRNA confirmed the pivotal role of the NRF2 pathway in the anti-inflammatory mechanism of apomorphine. Indeed, *Nfe2l2* siRNA did not only decrease the expression of *Nfe2l2* and its target genes (Fig. 11) but also restored the inflammatory conditions linked to the presence of A53T and the subsequent microglial reactivity (Fig. 12). As discussed above, this confirms the interaction between NRF2 and NFĸB signalling pathways. Our results are in accordance with Malhotra and collaborators (Malhotra et al. 2010), who established that NRF2 signalling pathway can regulate more than 600 genes, of which more than 200 encode cytoprotective proteins associated with inflammatory and neurodegenerative diseases (Papp et al. 2012; Hayes and Dinkova-Kostova 2014; Ahmed et al. 2017).

Unlike R-apomorphine which is a dopamine receptor agonist, the S-isomer has, to our knowledge, no known receptor. However, we have described that both enantiomers are equally potent in terms of NRF2 pathway activation. This is in accordance with the study reported by Mead and collaborators (Mead et al. 2013). Based on our results, both enantiomers of apomorphine showed the same effects on the A53T-induced primary microglia reactivity. Indeed, S-Apo but also R-Apo reduced microglial reactivity by a unique NRF2-dependent mechanism. This was confirmed by the use of trigonelline but also by a gene silencing approach. It is important to note that, even if the effects on microglial reactivity are similar for both enantiomers, we were able to highlight some differences in terms of MAPK activation. These disparities on MAPK phosphorylation status might indicate that the R-isomer might partially bind to the dopamine receptors. It has indeed been reported that the activation of dopaminergic receptors leads to the activation of MAPK pathway by especially increasing ERK phosphorylation (Li et al. 2006; Cahill et al. 2014; Mariani et al. 2019; Wang et al. 2020) resulting in a subsequent STAT1 phosphorylation (Kim et al. 2002; Tichauer et al. 2007; Song et al. 2017). The differential MAPK and JAK-STAT signaling pathways responses seem then to be independent of the recruitment of the NRF2 transcription factor. Finally, the R-Apo impact is likely linked to its antioxidant property to activate the NRF2 pathway rather than to its binding to dopamine receptors.

## Conclusion

Taken together, our work described how apomorphine treatment modulates microglial activation. Using enantiomers of apomorphine, we showed that R-Apo, a dopamine agonist, but also the S-Apo, which lacks dopamine receptor agonist activity, similarly decreased the microglial reactivity induced upon an A53T exposure. In our in vitro experimental conditions, apomorphine treatment rapidly decreased microglial reactivity. As underlying mechanism, we showed that activation of the NRF2/ARE pathway decreased the A53T mutant α-synuclein-induced microglial reactivity. The recruitment of the NRF2 transcription factor regulates the cellular redox status (*Hmox1* and *Nqo1* overexpression), decreases the overexpression of pro-inflammatory genes as well as the release of pro-inflammatory mediators and finally restores the microglial phagocytosis capacity. Known to be a critical transcriptional activator for antioxidant responses, NRF2 also appeared to be a powerful modulator in the course and/or outcome of inflammatory diseases and neurodegenerative diseases (Staurengo-Ferrari et al. 2018; Bahn and Jo 2019). Although our results may be pertinent to neurodegenerative diseases involving inflammation, further experiments are needed to better understand the importance of the KEAP1-NRF2-ARE pathway in the brain and its potential therapeutics impact.

## Abbreviations

α-syn: Alpha-synuclein
α-Tub: Alpha-tubulin
AP-1: Activator protein 1
Apo: Apomorphine
ARE: Antioxidant response element
cAMP: Cyclic adenosine monophosphate
CNS: Central Nervous System
ERK: Extracellular regulated kinase
HDAC1: Histone deacetylase 1
*Hmox*: Heme oxygenase
HRP: Horseradish peroxidase
KEAP1: Kelch-like ECH-associated protein 1
MACS: Magnetic cell sorting
MAPK: Mitogen-activated protein kinase
NFĸB: Nuclear factor kappa B
*Nfe2l2*: Nuclear factor E2-related factor 2 (protein coding gene)
*Nqo1*: NAD(P)H quinone oxidoreductase 1
NRF2: Nuclear factor E2-related factor 2 (protein)
PD: Parkinson’s disease
PKA: Protein kinase A
R-Apo: R-apomorphine
RNS: Reactive nitrogen species
ROS: Reactive oxygen species
S-Apo: S-apomorphine
siRNA: Small interfering RNA
SNpc: Substantia Nigra pars compacta
STAT: Signal transducer and activator of transcription
tBHQ: Tertiary butylhydroquinone
WT: Wild-type
